# Secondary prophylactic treatment and long‐term prognosis after TIA and different subtypes of stroke. A 25‐year follow‐up hospital‐based observational study

**DOI:** 10.1002/brb3.603

**Published:** 2016-11-28

**Authors:** Sven‐Erik Eriksson

**Affiliations:** ^1^ Division of NeurologyDepartment of MedicineFalun HospitalFalunSweden

**Keywords:** anticoagulants, ASA, myocardial infarction, predictors, recurrent stroke, survival

## Abstract

**Objectives:**

To assess long‐term prognosis after transient ischemic attack (TIA)/subtypes of stroke relative to secondary prophylactic treatment(s) given.

**Materials and Methods:**

Retro/prospective follow‐up of patients hospitalized in the Stroke Unit or in the Department of Neurology, Linköping, in 1986 and followed up to Feb. 2011.

**Results:**

A total of 288 men were followed up for 2254 years (mean 7.8 years) and 261 women for 1984 years (mean 7.6 years). In men, the distribution to anticoagulants (AC) (warfarin treatment) was 18%, antiplatelet therapy (APT) usually ASA 75 mg/day 54%, untreated 27%, unknown 2%. In women, the distribution to AC was 15%, APT 60%, untreated 23%, unknown 2%, respectively. Mortality rates at 1 year, 10 years, and 25 years for men were 21%, 67%, and 93%, respectively, versus the rates in women of 24%, 71%, and 90%, respectively. Survival curves showed markedly increased risk of death compared to the normal population. AC treatment was more favorable for men regarding the annual risk of stroke, compared with APT (9.4% vs. 9.8%), as well as the risks of MI, (5.6% vs. 6.7%), and death (8.1% vs. 10.3%), compared to women for stroke (11.6% vs. 8.8%) and MI (5.3% vs. 3.7%) but not for death (8.3% vs. 8.4%). The risk of fatal bleeding was 0.86% annually on AC compared to 0.17% on APT. According to Cox regression analysis included patients with TIA/ischemic stroke, first‐line treatment had beneficial effects on survival: AC OR 0.67 (0.5–0.9), APT 0.67 (0.52–0.88) versus untreated.

**Conclusions:**

Patients with a history of TIA/stroke had a higher mortality rate versus controls, providing support for both primary and secondary prophylaxis regarding vascular risk factors for death. This study also provided support for secondary prophylactic treatment with either AC or ASA (75 mg once daily) to reduce the vascular risk of death unless there are contraindications.

## 1 Introduction

The risk of stroke after a transient ischemic attack (TIA) is high especially during the first 3 months and figures of 10–20% have been reported (Coull, Lovett, & Rothwell, 2004; Eliasziw, Kennedy, Hill, Buchan, & Barnett, 2004; Eriksson & Olsson, 2001; Hill et al., 2004; Purroy et al., 2007). Similar results have been reported for minor, moderate stroke patients (Coull et al., 2004) or considerably lower figures (Eliasziw et al., 2004; Eriksson, & Link, 1983). However, after 14–years follow up a cumulative risk of 63.2% (CI 55.6–71) of having a recurrent stroke was reported in a previous study that included different subgroups of stroke patients (Eriksson & Olsson, 2001).

Atrial fibrillation (AF) is an important risk factor for cardiac embolism, especially in combination with other risk factors, with a high risk of severe stroke and/or recurrence (Friberg, Benson, Rosenqvist, & Lip, 2012; Goto et al., 2008; Kim et al., 2011). In primary or secondary prophylactic treatment of patients with AF, AC and APT have each been shown to be a better alternative than a placebo, with warfarin better than APT but with an increased risk of bleeding (Alberts, Eikelboom, & Hankey, 2012; Blackshear et al., 1996; Connolly et al., 2008; Fuster et al., 2006; Hart, Pearce, & Aguilar, 2007; Hylek et al., 2003; Laupacis et al., 1994). Novel oral anticoagulants (NOACs) have been reported to have the same effects or to perform even better than warfarin with an equal or lower risk of major bleeding (Alberts et al., 2012; Hankey, 2014; Hori et al., 2013; Lopes et al., 2012), and NOACs have shown large differences in their favor regarding the risk of stroke or systemic embolism, compared with ASA (Alberts et al., 2012; Diener et al., 2012).

Several randomized trials using secondary prophylactic treatment, either with AC or APT or conducting comparisons between AC and APT after TIA/stroke due to arterial thromboembolism have found APT to have the same effects as AC, or APT has been deemed to be a better option because of a lower risk of major bleeding or other factors (Antithrombotic Trialists' Collaboration, 2009; Campbell, Smyth, Montalescot, & Steinhubl, 2007; De Schryver, Algra, Kappelle, van Gijn, & Koudstaal, 2012; Gouya et al., 2014; Hankey, 2014; Lemmens, Chen, Ni, Fieuws, & Thijs, 2009; Maasland et al., 2009; Sandercock, Counsell, Tseng, & Cecconi, 2014). Urgent secondary prophylactic treatment(s) decrease the risk of (recurrent) stroke considerably after TIA or minor stroke due to arterial thromboembolism (Rothwell et al., 2007). However, the risk of recurrent stroke did not decrease over time with APT in patients with arterial embolisms (Lemmens et al., 2009). A higher dose of aspirin than 75–100 mg once daily explained the increased risk of side effects, but it did not provide any better protection regarding the outcomes of cardiovascular events (Campbell et al., 2007).

Patients with either TIA and/or an ischemic or hemorrhagic stroke have besides an increased risk of suffering (recurrent) stroke, increased risks of experiencing myocardial infarction (MI) or a vascular cause of death over long‐term observation (Appelros, Gunnarsson, & Terent, 2011; Brønnum‐Hansen, Davidsen, & Thorvaldsen, 2001; Burns et al., 2011; Dhamoon, Sciacca, Rundek, Sacco, & Elkind, 2006; Eriksson & Olsson, 2001; Hardie, Hankey, Jamrozik, Broadhurst, & Anderson, 2004; Touze et al., 2005). Warfarin alone or in combination with aspirin was superior to aspirin alone regarding endpoints, but it increased the risk of major, nonfatal bleeding after MI (Hurlen, Abdelnoor, Smith, Erikssen, & Arnesen, 2002). In fact, the long‐term effects of administering AC/APT after TIA/stroke due to arteriosclerotic disease for the remainder of the patient′s life remains unknown (Cleland, 2006).

The present observational trial included patients with TIA/stroke hospitalized in the Stroke Unit or in the Department of Neurology in 1986. The purpose of this trial was to report the long‐term prognosis after TIA/subtypes of stroke relative to secondary prophylactic treatment(s) administered or not over a long‐term observation period, and to determine predictors of stroke, MI and death.

## 2 Materials and Methods

This study enrolled 549 patients, of whom 362 patients (66%) (M 171, W 191) were living in the Linköping area, and 187 (34%) (M 117, W 70) lived outside the Linköping area. A total of 370 patients (67%) (M 169, W 201) were hospitalized in the Stroke Unit and 179 patients (33%) (M 119, W 60) on the general ward of the Department of Neurology. Ninety‐four patients (M 45, W 49) were excluded from this trial, of whom 55 were hospitalized in the Department of Neurology. Causes for exclusion were the following: epilepsy in 10 cases, cerebral contusion in 3, subarachnoid hemorrhage in 8, multiple sclerosis in 3, brain tumor in 5, living abroad or no records found in 6, transient global amnesia in 8, >3 months from onset of latest TIA/stroke to admission in 13, and other causes in 38 cases. No patients were lost to follow‐up over the observation period.

TIA was defined as acute onset of focal neurological symptom(s) and sign(s) or retinal symptoms that were transient within 24 hr. The diagnosis of MI was assessed by electrocardiogram (ECG), usually with elevated cardiac enzymes, and the patients usually had ischemic symptoms, or the diagnosis was verified by morphological findings. The criteria for other diagnoses and classifications were provided in a previous study (Eriksson & Olsson, 2001). Cause of death was based on the underlying cause defined by the World Health Organization (WHO) as the disorder that started a chain of events leading to death.

Previous study had reported predictors of stroke, and death during long term observation time, but the using of secondary prophylactic treatment only in the acute phase (Eriksson & Olsson, 2001). The aim of this study was to report the long‐term prognosis for TIA and different subgroups of stroke with a focus on documenting events of strokes, myocardial infarctions, deaths, depending on the diagnosis, sex and type of secondary prophylactic treatment(s) (AC, APT) administered or not during the observation time. Other aims of the study was to report other factors that could be of importance for the long‐term course: such as the occurrence of cancers in this cohort of patients, occurrence of atrial fibrillation on ECG (not known before), prescription of lipid‐lowering drugs/ACE inhibitor or angiotensin‐II‐receptor blockers.

The study began in 2003 after approval by the Ethics Committee of the University Hospital in Linköping, and a completed application was filed in 2008. The results reported in this trial were based on data reported previously (Eriksson & Olsson, 2001), as well as new data supplied in medical records from different clinics, general practitioners, cancer registries, death certificates, and autopsy reports, which were all scrutinized, and from complete telephone interviews and/or (in a few cases letters to) patients or their relatives/caregivers six times since 2003. All of the included patients were followed up to Feb. 2011

### 2.1 Anticoagulant/antiplatelet therapy

Patients were treated after admission either with AC (usually in combination with heparin for the first few days) or with APT, which usually consisted of half a tablet of Albyl ^®^ Minor (Recip) 250 mg given once daily, except patients with reduced consciousness/severe disability and/or e.g. history of a bleeding disorder. For each patient, the benefit/risk profile assessment was made before the choice of treatment, or no treatment after having ruled out another cause of the patient's illness. The results from the analysis of International Normalized Ratio (INR) values among the AC‐treated patients have not been reported, but the recommendation was that the INR values should be 2–3. After 1990, half a tablet of Albyl ^®^ Minor was replaced by Trombyl ^®^ 75 mg once daily. Events of strokes, myocardial infarctions, and deaths were classified according to the treatment(s) given. Short interruption(s) of treatment due to surgery or other reasons inside or outside the hospital did not change this classification of treatment. Procedure‐related events of strokes/MIs were classified by the secondary prophylactic treatment that was given before the surgery. Fatal index strokes were reported at the latest treatment, whereas fatal recurrent strokes were classified as either AC or APT given or both or untreated at the time of the event of stroke even if >1 month had elapsed from ictus to death. This classification was also applied in patients with other causes of death if secondary prophylactic treatment was stopped due to a serious disease. Of patients treated with AC plus APT included only AC in the observation period unless otherwise stated. The study reports events of strokes, MIs, deaths per 100‐years of observation time. Intention to treat analysis included all of the patients with TIA/ischemic stroke.

### 2.2 Statistical Analysis

Statistical analysis was performed using IBM SPSS software for Windows (Statistical Package for the Social Sciences; SPSS Inc., version 20). Student's t‐test, the *X* ^2^‐test corrected with Bonferroni's methods, and the Mann–Whitney U‐test were used to compare data between groups. The Kaplan–Meier life table technique was used to estimate the risk of first event of stroke, MI, and the surviving proportion, and the log rank test was used to analyze the differences in occurrences of events between subgroups. A Cox proportional hazards regression model, analyzing different risk factors/first‐line treatments was used to assess the odds ratios (ORs) of recurrent stroke, MI, and death. Age is a continuous variable with a linear increase in risk each year. Systolic/diastolic blood pressure was a continuous variable with a linear increase in risk per mm Hg increase in blood pressure.

## 3 Results

The baseline characteristics of the 549 included patients, of whom 261 patients (48%) were women, are shown in Table 1. Among patients admitted from the catchment area of Linköping 53% of all ischemic strokes occurred in women, of which 29% were cardioembolic (CE) cerebral infarctions, compared to 19% in men (*p *= .056). Among women with CE, 72% were older than 74 years old vs. 59% in men (ns). The corresponding distributions of intracerebral hemorrhage (ICH) were 55%, and 45%, respectively. Among women, 81% were older than 74 years old vs. 31% in men (ns). Approximately, one‐fourth of the included men admitted from the catchment area of Linköping with stroke had a history of a previous stroke versus one fifth of the women. Among the patients admitted due to TIA, 38% of the men had remaining symptoms and signs at admission, compared with 18% of the women. In total, 15% of the men and 13% of the women had TIAs <3 months before the latest symptoms of TIA/stroke (before stroke 10% and 11%, respectively). There were no statistically significant differences between sexes from onset of acute neurological symptoms and signs and admission (men, mean 3.1 days, std. 11.8, median 0, women 2.8; 11.5; 0). Among men and women, 11% and 9%, respectively, had been hospitalized for more than one day at another hospital/clinic before admission. CT ‐scans were performed in 77% of men and 67% of women. In patients with TIA/atherosclerotic cerebral infarction (ACI) with symptoms within the carotid artery supplying area, doppler/angiography was performed in 81 men (51%) versus 65 women (48%). Greater than 50% stenosis/occlusion of the symptomatic internal carotid artery was found in 33% of men versus 23% in women (ns).

**Table 1:** Baseline characteristics

Diagnosis	TIA		ACI		CE		LI		ICH		M	*p*‐value	*F*	*p*‐value	M vs. F*p*‐value
Sex	M	F	M^a^	F^b^	M^c^	F^d^	M^e^	F^e^	M	F					
n, (%)	34, (11.8)	17, (6.5)	152, (52.8)	143, (54.8)	41, (14.2)	54 (20.7)	35, (12.2)	23, (8.8)	26, (9)	24, (9.2)	288		261		ns
Age, years, range	42–83	51–90	39–91	36–92	58–90	60–96	48–83	41–88	39–81	47–89	39–91		36–96		
Mean, median	64, 62	70.1, 67	70.3, 71.5	69.9, 71	75.3, 76	78.3, 79	69.9, 71	70.9, 73	64.1, 65.5	74.6, 76	69.7, 71	.000	72.2. 74	.000	.009
≤74^f^	26 (14)	11 (2)	95 (47)	88 (37)	19 (8)	17 (4)	26 (9)	12 (1)	21 (12)	6 (3)	187 (90)		134 (47)		
≥75^f^	8 (3)	6 (1)	57 (15)	55 (12)	22 (6)	37 (4)	9 (2)	11 (1)	5 (1)	18 (5)	101 (27)		127 (23)		
Stroke, (%)															
Minor stroke			50	51	34	30	57	70	8	0	44		43		
Moderate stroke			21	20	17	15	34	22	11	12	21	.000	18	.000	ns
Major stroke			29	29	49	56	9	9	81	87	35		39		
Lowered level of consciousness, (%)			2	4	10	7			27	54	5	.000	9	.000	ns
Progressive stroke, (%)			14	13	17	7	14	4	4	8	13	ns	10	ns	ns
Territory^g^, %															
Carotid left hemisphere	47	65	49	41	50	45	40	56	65	42	50		45		
Vertebrobasilar territory	12	24	16	16	7	9	3	0	4	12	11	ns	13	ns	ns
Blood pressure at admission, mm Hg	158.2 (32.1)	176.8 (32.4)	163.5 (26.9)	168.4 (32)	158.9 (29.6)	166.8 (23.5)	170.4 (32.4)	185.9 (35)	176.5 (29.1)	177.5 (35.8)	164.2 (29.1)	ns	171 (31.4)	ns	.009 (syst)
Systolic (SD) diastolic (SD)	93.1 (13.4)	97.1 (14.5)	90.1 (12.8)	90.6 (12)	91.1 (16.4)	90.6 (13.3)	90.4 (12.1)	96.3 (14.1)	97.3 (15)	93.2 (11.8)	91.3 (13.6)	ns	91.7 (12.7)	ns	
Blood pressure at admission^h^, mm Hg															
Systolic (SD)	164.4 (33.1)		165.9 (29.6)		163.4 (26.5)		176.5 (34)		177 (32.2)						
Diastolic (SD)	94.4 (13.7)		90.3 (12.4)		90.8 (14.6)		92.8 (13.1)		95.4 (13.7)						
Fasting blood glucose, mean, (SD)	5.7 (2.7)	5.6 (1.3)	6.5 (2.8)	7.0 (3.1)	6.8 (3.2)	7.3 (3.3)	6.1 (3)	5.8 (1.5)	6.6 (2.2)	8.8 (3.2)	6.4 (2.8)	ns	7 (3)	.01	.044
Missing value,%	6	6	7	8	7	6	3	9	15	29	7		9		
Treatments, %															
Diuretics	23	47	41	42	66	76	26	48	27	33	40	.000	49	.000	.025
β– Blockers	12	35	22	27	17	28	23	26	38	17	22	ns	27	ns	ns
Calcium channel blockers	6	6	10	10	12	28	6	4	8	12	9	ns	13	.014	ns
ACE–inhibitor	3	0	3	3	2	0	0	0	0	0	2	ns	2	ns	ns
First–line treatment^i^, %															
AC	76	41	42^i^	38	24	35	51	30			41^i^		33		
APT	21	53	33^i^	36	34	39	40	57			30^i^		36		
Untreated	3	6	26	27	41	26	9	13	100	100	30		31		ns
CT–scan, %	*n *= 21	*n *= 7	*n *= 114	*n *= 95	*n *= 27	*n *= 35	*n *= 33	*n *= 21			*n *= 195		*n *= 158		
Visible infarction, %															
No	86	86	29	37	26	31	18	24			33	.000	36	ns	ns
One	14	14	41	44	30	43	27	38			34		42		
Multiple	0	0	30	19	44	26	55	38			33		22		
Visible LI	5	14	18	15	22	14	64	67			25	.000	22	.000	ns
Leukoaraiosis	10	43	18	22	33	23	27	14			21	ns	22	ns	ns
Previous TIA, %	62	47	23	18	5	20	9	22	0	8	21	.000	20	.037	ns
>90 days ago, %	21	29	9	5	0	11	3	13	0	8	7	.004	9	.014	ns
Previous stroke, %	15	29	31	22	46	22	23	17	23	21	30	.032	22	ns	ns
History of angina pectoris, %	18	6	19	10	17	13	17	13	4	0	17	ns	10	ns	.018
History of myocardial infarction or pathological Q waves on ECG, %	15	6	32	18	27	20	20	13	11	13	26	ns	17	ns	.013
Heart failure, %	3	18	27	29	83	85	20	26	19	29	31	.000	40	.000	.025
AF on ECG, %	3	6	2	0.8	90	87	0	9	18	32	17	.000	23	.000	ns
Missing ECG, %	3	0	9	8	0	0	0	0	15	21	7	–	6	–	–
History of claudication intermittens, %	9	0	7	4	7	9	6	0	0	0	7	ns	4	ns	ns
Hypertension^j^, %	53	71	67	73	76	93	63	78	65	67	66	ns	77	.031	.006
History of diabetes, %	12	18	22	24	17	20	20	13	4	8	18	ns	20	ns	ns
Fasting blood glucose ≥6.1 mmol/L or history of diabetes, %	21	23	38	50	51	44	34	39	42	62	38	ns	48	ns	.025
Fasting S‐cholesterol^k^ , %															
<5 mmol/L	20	10	15	13	18	25	26	0	29	33	18		13		
5–6.4 mmol/L	40	50	52	43	18	42	37	45	29	33	43	ns	44	ns	ns
≥6.5 mmol/L	40	40	33	43	64	33	37	54	43	33	38		43		
Missing,%	41	41	56	58	73	78	46	52	73	87	57	–	63	–	–
Fasting S‐triglycerides^l^, %															
<1.6 mmol/L	60	50	52	53	45	58	44	54	100	100	23	ns	55		
1.6–2.2 mmol/L	20	40	22	18	27	17	39	18	0	0	10		20	ns	ns
≥2.3 mmol/L	20	100	25	28	27	25	17	27	0	0	9		25		
Missing, %	41	41	56	58	73	78	49	52	73	87	57	–	63	–	–

a No CT‐scan/autopsy in 35 cases.

b No CT‐scan/autopsy in 44 cases.

c No CT‐scan/autopsy in 13 cases.

d No CT‐scan/autopsy in 17 cases.

e No CT‐scan/autopsy in 2 cases.

f Number within parenthesis denotes patients living outside the Linköping catchment area.

g In 3 cases ,the territory was unknown.

h *p*‐value between diagnoses, systolic blood pressure, *p *= .01, diastolic blood pressure, *p *= .043.

i In one case, both treatments.

j Hypertension/treatment with antihypertensive drugs.

k Fasting cholesterol value, mean (SD), men 124 cases, 6.2 (1.4); women 96 cases, 6.3 (1.3).

l Fasting triglycerides, mean (SD), men 123 cases, 1.8 (1.1); women 96 cases, 1.7 (0.9).

### 3.1 Outcomes during the first month

Progressive stroke occurred in 13% of the patients with ischemic stroke in men (carotid territory [CT] 29/vertebrobasilar territory [VB] 4, [minor 18, moderate 7, major 8]), of which 10 cases were fatal, compared with 23 cases of women (10%) (CT 20, VB 3 (minor 13, moderate 3, major 7), 1 of which was fatal (*p *= .016). The fatality rate for patients with ICH was 40% (men 35% vs. women 46%), which was statistically significantly higher compared with the other diagnoses in men and women (*p *< .05): CE 21% (27% vs. 13%); and (ACI) 10%, (11% vs. 9%). Thirty of 37 patients with lowered levels of consciousness at admission died (ICH 16, CE 6, and ACI 8). Among all of the deaths in men, the distribution of the severity of the stroke at admission was a minor stroke in six cases, a moderate stroke in 3, and a major stroke in 27, compared with 1, 1, and 29 cases in women, respectively.

In total 19 patients (4%) suffered a (recurrent) stroke: 9 men versus 10 women. The occurrence of stroke per 100 patient‐years of observation time for men on AC was 33%, compared to 44% for men on APT, and 54% in untreated men versus 62%, 38%, and 76%, respectively in women on AC, women on APT and untreated women, given total annual risks on “treatment” of 45%, 41% and 65%, respectively.

### 3.2 Events of strokes and myocardial infarctions

**Figure 1:**
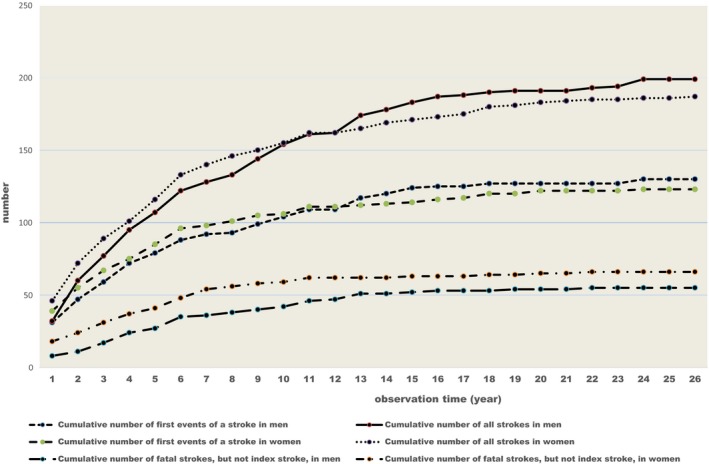
The cumulative number of first events of a stroke, all events of strokes and fatal strokes among men and women during the observation period

Kaplan‐Meier estimates of the probabilities of having a stroke for each diagnosis during the whole observation time in men and women are shown in Table 2. In total, 130 men (45%) suffered 199 strokes (55 fatal), compared with 123 women (47%) who had 187 strokes (66 fatal) (Figure 1) Eighty‐three (42%) unspecified strokes occurred in men versus 90 (48%) in women (Table 3).

**Table 2:** Kaplan–Meier estimates of probabilities of first event of stroke after admission in men and women, 1986–2011

Stroke	TIA	ACI	CE	LI	ICH	Total
	Cumulative risk %, 95 % CI	Cumulative risk %, 95 % CI	Cumulative risk %, 95 % CI	Cumulative risk %, 95 % CI	Cumulative risk %, 95 % CI	Cumulative risk %, 95 % CI
Men						
1 month	2.9 (0–8.9)	5.3 (1.3–9.3)	0	0	0	3.1 (1.1–5.1)
3 months	2.9 (0–8.9)	7.4 (3.4–11.4)	3.3 (0–9.3)	2.9 (0–7.9)	0	5.1 (2.8–8.1)
6 months	8.8 (0–18.8)	9.7 (4.7–14.7)	10.2 (0–22.2)	2.9 (0–7.9)	0	7.9 (4.9–10.9)
1 year	11.8 (0.8–22.8)	13.6 (7.6–19.6)	20.6 (5.6–35.6)	2.9 (0–7.9)	5.9 (0–16.9)	12 (8–16)
2 years	14.7 (2.7–36.7)	22.7 (15.7–29.7)	31.9 (14.9–48.9)	8.6 (0–17.6)	5.9 (0–16.9)	19.4 (14.4–24.4)
5 years	30.5 (14.5–46.5)	34.4 (26.4–42.4)	43.9 (24.9–62.9)	36 (20–52)	12.2 (0–28.2)	33.6 (27.6–39.6)
10 years	41.5 (24.5–58.5)	46.8 (36.8–56.8)	72 (52–92)	53.1 (34.1–72.1)	41.4 (12.4–70.4)	49.3 (42.3–56.3)
15 years	64 (45–83)	63.9 (52.9–74.9)	90.7 (74.7–100)	73.2 (53.2–93.2)	53.2 (22.2–84.2)	67.4 (55.4–75)
20 years	64 (45–83)	74.7 (61.7–87.7)	90.7 (74.7–100)	73.2 (53.2–93.2)	53.2 (22.2–84.2)	71.8 (63.8–79.8)
Total	73 (52–94)	79.8 (65.8–93.8)	90.7 (74.7–100)	73.2 (53.2–93.2)	76.6 (40.6–100)	78.9 (69.9–87.9)
Women						
1 month	5.9 (0–16.9)	4.2 (1.2–7.2)	3.7 (0–9.7)	4.3 (0–12.3)	0	3.8 (1.8–5.8)
3 months	5.9 (0–16.9)	8 (3–13)	12.3 (3.3–21.3)	8.7 (0–20.7)	0	8.1 (5.1–11.1)
6 months	5.9 (0–16.9)	12 (7–17)	14.6 (4.6–24.6)	8.7 (0–20.7)	0	10.8 (6.8–14.8)
1 year	5.9 (0–16.9)	21.7 (14.7–28.7)	14.6 (4.6–24.6)	13.3 (0–27.3)	0	16.8 (11.8–21.8)
2 years	5.9 (0–16.9)	29.9 (21.9–37.9)	28.8 (15.8–41.8)	13.3 (0–27.3)	0	24.4 (18.4–30.4)
5 years	5.9 (0–16.9)	47.4 (38.4–56.4)	49.2 (34.2–64.2)	18.4 (2.4–34.4)	9.1 (0–26.1)	39.5 (32.5–46.5)
10 years	13.7 (0–31.7)	60.6 (51.6–69.6)	60.9 (44.9–76.9)	29.2 (9.2–49.2)	48.1 (13.1–83.1)	52.9 (45.9–59.9)
15 years	31 (5–57)	65.3 (55.3–75.3)	60.9 (44.9–76.9)	42.1 (19.1–65.1)	65.4 (29.4–100)	60 (52–68)
20 years	60.6 (31.6–89.6)	71.1 (61.1–81.1)	80.4 (52.4–100)	51.8 (25.8–77.8)	65.4 (29.4–100)	69.4 (61.4–77.4)
Total	70.4 (52.4–98)	71.1 (61.1–81.1)	80.4 (52.4–100)	51.8 (25.8–77.8)	65.4 (29.4–100)	70.9 (62.9–78.9)

Outcomes over the long term were significantly different among included subgroups (log rank test): menTIA vs. CE, *p *= .036; CE vs. ICH, *p *= .013; women TIA vs. CE, *p *= .012; ACI vs. LI, *p *= .040; CE vs. LI, *p *= .042. No differences between diagnosis and sex and in total between sexes. Compared overall strata, TIA vs. CE, *p *= .001; LI vs. CE, *p *= .008; ACI vs. ICH, *p *= .035; CE vs. ICH, *p *= .004.

**Table 3:** Territory of new neurological symptoms and signs compared with territory at admission and whether any CT‐scan/autopsy was performed or not. Numbers within parenthesis denotes fatal strokes

Number of stroke	1		2		3		4		5		6		7	
Sex	M	F	M	F	M	F	M	F	M	F	M	F	M	F
Same territory as index symptoms + investigation	34 (3)	26 (8)	14 (3)	14 (1)	2	4 (1)	2		1		1 (1)			
Same territory as index symptoms + no investigation	28 (8)	25 (7)	11 (5)	12 (9)	4 (3)	1	1	3		1		1		
Another territory as index symptoms + investigation	36 (2)	31 (8)	10 (4)	8 (1)	4 (1)	2		2		1		1		
Another territory as index symptoms + no investigation	20 (8)	21 (6)	5 (2)	5 (4)		1	1 (1)	1 (1)						1
Uncertain territory	5 (3)	13 (11)	5 (5)	4 (4)	2 (1)		1	1 (1)						
SAH/ICH	7 (2)	7 (3)	2 (2)	1 (1)	3 (1)									
Total^a^	130^b^ (26)	123^c^ (43)	47^d^ (21)	44^e^ (20)	15^f^ (6)	8^g^ (1)	5^h^ (1)	7^i^ (2)	1	2	1 (1)	2	0	1

First event, mean (95% confidence interval [CI], median (95% CI) days. Men, same territory, *n *= 62, 1540 (1089–1992), 846 (349–1342); another territory, *n *= 56, 2232 (1692–2773), 1377 (575–2178), uncertain territory, *n *= 5, 3057 (1502–4612), 4006 (0–9274), ICH/SAH, *n *= 7, 1942 (1606–2278), 1254 (947–1560). Women, same territory, *n *= 51, 1225 (766–1885), 519 (186–852), another territory, *n *= 52, 1825 (1296–2354), 1186 (719–1652), uncertain territory, *n *= 13, 1312 (251–2371), 611 (0–1529), ICH/SAH, *n *= 7, 2703 (1222–4184), 2087 (1517–2657) .

^a^Range, mean, median, SD (days).

^b^1–6317, 1789, 1249, 1691, ^c^4–7033, 1467, 853, 1691, ^d^177–7853, 2726, 2440, 1900, ^e^16–7421, 1879, 1506, 1781, ^f^444–8131, 2841, 2628, 2163, ^g^36–4689, 2162, 1547, 1939, ^h^600–8579, 3856, 1053, 4057, ^i^1143–7805, 4825, 5374, 2436.

In men, the recurrence of TIA was reported in 47% compared to 35% in women, whereas the occurrence of TIA among stroke patients was considerably lower: in men, 12%; in women, 8%. TIA + stroke occurred in 12% of men versus 6% of women, whereas new events of either TIA and/or stroke occurred in 50% of men versus 51% of women. Stroke due to carotid surgery occurred in one man with a history of TIA and in 2 women with histories of ACI. In total, eight men had strokes <6 months after MI (<30 days, 4; >3 months, 2), and 5 women had had 6 strokes (<30 days in 5 cases). During the observation period, 53 men had had paroxysmal or permanent AF on ECG (not known before), and in total, 33 of these patients had 53 strokes, of whom 19 patients had 24 strokes after registered AF, whereas 20 of 30 women had 27 strokes, of whom 16 women had 19 strokes after recorded AF.

**Figure 2:**
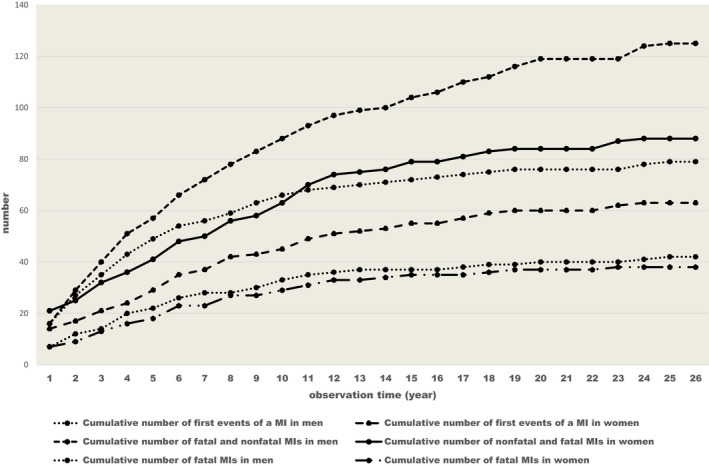
The cumulative number of first events of myocardial infarction, all events of myocardial infarctions and fatal myocardial infarctions among men and women during the observation period

Kaplan–Meier estimates were calculated of the probabilities of having had an MI for each diagnosis over the whole observation periods in men and women (Table 4). In total, 79 men (27%) had 125 MIs, of which 42 were fatal (Figure 2) (first, 29; second, 9; third, 3; and fourth, 1). Among women, 63 patients (24%) had 88 MIs, of which 38 were fatal (Figure 2). Forty‐six patients had one MI (fatal, 24), 13 patients had 2 MIs (fatal, 11), and a fatal MI occurred in each group in those with 3, 4, and 5 MIs.

**Table 4:** Kaplan–Meier estimates of probabilities of first event of myocardial infarction after admission in men and women, 1986–2011

Myocardial infarction	TIA	ACI	CE	LI	ICH	Total
	Cumulative risk, 95 % CI	Cumulative risk, 95 % CI	Cumulative risk, 95 % CI	Cumulative risk, 95 % CI	Cumulative risk, 95 % CI	Cumulative risk, 95 % CI
Men						
1 month	0	0.7 (0–2.1)	5.6 (0–13.2)	0	0	1.1 (0–2.2)
3 months	0	1.4 (0–3.4)	5.6 (0–13.2)	0	0	1.5 (0–3)
6 months	0	5.3 (1.6–9)	5.6 (0–13.2)	2.9 (0–8.4)	0	3.9 (1.5–6.2)
1 year	0	8.5 (3.8–13.2)	9 (0–18.8)	2.9 (0–8.4)	5.9 (0–17.1)	6.4 (3.3–9.5)
2 years	8.9 (0–18.5)	14.5 (8.2–20.8)	9 (0–18.8)	5.7 (0–13.3)	5.9 (0–17.1)	11.3 (7.2–15.4)
5 years	22.9 (7.8–38)	28.6 (20–37.2)	9 (0–18.8)	19.7 (5.4–3)	5.9 (0–17.1)	22.9 (17.2–28.5)
10 years	35.1 (17.1–53.1)	42.8 (32.6–53)	16.6 (0–33.4)	23.6 (8.1–39.1)	17.6 (0–41.3)	35 (27.7–42.2)
15 years	46.1 (25.7–66.5)	46.7 (35.9–57.5)	16.6 (0–33.4)	47.9 (15.2–80.6)	17.6 (0–41.3)	41.9 (33.5–50.3)
20 years	58.1 (36.5–79.7)	53.1 (40.4–65.8)	16.6 (0–33.4)	47.9 (15.2–80.6)	17.6 (0–41.3)	49.3 (39.3–59.3)
Total	65.1 (43.1–87)	78.4 (47.6–100)	16.6 (0–33.4)	47.9 (15.2–80.6)	17.6 (0–41.3)	65.3 (43.6–87)
Women						
1 m	0	1.5 (0–3.5)	0	0	0	0.8 (0–2)
3 m	5.9 (0–17.1)	3.1 (0.2–6)	0	0	0	1.7 (0–3.5)
6 m	5.9 (0–17.1)	4.7 (1–8.4)	2.4 (0–7.1)	9.1 (0–21)	0	4.5 (1.7–7.2)
1 year	5.9 (0–17.1)	8.1 (3.4–12.8)	2.4 (0–7.1)	9.1 (0–21)	0	6.4 (3.1–9.7)
2 years	5.9 (0–17.1)	9.8 (4.5–15.1)	5.4 (0–12.8)	9.1 (0–21)	0	8 (4.3–11.7)
5 years	19.3 (0–39.1)	17 (9.9–24)	17.8 (3.1–32.5)	9.1 (0–21)	0	15.2 (10.1–20.3)
10 years	19.3 (0–39.1)	32.2 (22.2–42.2)	29.7 (9.7–49.7)	15.6 (0–32.)	16.7 (0–46.5)	28.3 (20.8–35.7)
15 years	34.3 (9.4–59.2)	47.4 (35.2–59.5)	29.7 (9.7–49.7	26.1 (2–50.2)	16.7 (0–46.5)	40.4 (31.2–49.6)
20 years	34.3 (9.4–59.2)	53.7 (41.2–66.2)	29.7 (9.7–49.7)	36. 8 (48–64.9)	91.7 (80.7–100)	47.6 (37.6–57.6)
Total	34.3(9.4–59.2)	61.4 (48.3–74.5)	29.7 (9.7–49.7	36. 8 (48–64.9)	91.7 (80.7–100)	53.3 (42.5–64.1)

Outcomes over the long term were significantly different among included subgroups (log rank test): men, TIA vs. CE, *p *= .001; TIA vs. ICH, *p *= .003; ACI vs. CE, *p *= .007; ACI vs. ICH, *p *= .015; women; CE vs. ACI, *p *= .016; ACI vs. ICH, *p *= .027. No differences between diagnosis and sexes and in total between sexes. Pairwise pooled TIA vs. CE, *p *= .000; TIA vs. ICH, *p *= .000; ACI vs. CE, *p *= .000; ACI vs. ICH, *p *= .001, ICH vs. LI, *p *= .028.

Procedure‐related MIs occurred in 2 men after coronary artery bypass grafting (CABG) performed due to angina pectoris and in one woman after percutaneous coronary intervention (PCI) with a stent after MI. During the observation period, 12% of the patients had both a MI and a stroke event: 14% of men and 9% of women. For all patients at risk of having a recurrent stroke, MI, or both, the rate was 58% in men versus 62% in women.

### 3.3 Prescription of lipid‐lowering drugs/ACE inhibitors or angiotensin‐II‐receptor blockers and occurrence of malignant cancers

During the entire observation period, a total of 29 men (10%) and 19 women (7%) were treated with statins, whereas treatment either with ACE inhibitors or with angiotensin‐II‐receptor blockers (ARB) drugs, was administered to 63 men (22%) and 47 women (18%).

Sixty‐nine men had 80 malignant cancers (ICD‐10, C00‐C97), of which 22 had been diagnosed before the admission, compared with 74 malignant cancers in 71 women, of which 40 had been diagnosed before admission.

### 3.4 Death due to index stroke

Eighty patients died of index stroke (16%) 41 M [16%], 39 W[16%]; 42 patients (21 M, 21 W) died of stroke alone within one month, whereas the remaining patients died of complications due to stroke (ischemic stroke + pneumonia [8 M, 7 W], ICH + pneumonia [1 M, 1 W], ischemic stroke + pulmonary embolism [3 M, 5 W], ischemic stroke + pulmonary embolism + pneumonia [4 M, 2 W], ischemic stroke + sepsis/nephritis [2 W], or in combination with other diseases, such as ischemic stroke + MI (1 M) and ischemic stroke + heart failure (3 M, 1 W). In total, 10 deaths in men occurred more than one month after admission, compared with 11 deaths in women.

### 3.5 Death due to a recurrent stroke

The risk of death due to recurrent stroke was 25% in women versus 19% in men. The differences between sexes existed mainly for the first recurrent stroke: 65% versus 47%. In total, 36 of these deaths occurred within one month after the onset of stroke in men, compared with 44 in women. The causes of death in men were as follows: stroke alone in 19 cases; in combination with pneumonia in 22; sepsis in 3; MI in one; heart failure in 5 and other causes in 5 (status epilepticus 1, generalized arteriosclerosis 3, and chronic bronchitis 1). Among women, 34 patients died of stroke alone (3 ICH + 1 SAH), and the remaining patients died of complications due to stroke, such as pneumonia in 14 cases, pulmonary embolism in 4, MI in 3, heart failure in 2 and 9 from other causes (generalized arteriosclerosis 7, vomiting 1, suffocation 1).

### 3.6 Vascular and nonvascular cause of death

During the observation period 117 men (40.6%) died due to a vascular cause other than index stroke or a “recurrent stroke”: 40 died due to MI (14%); 55 died from cardio sclerosis, heart failure, or arrhythmia (19%); Eight died from gangrene, embolus not in the brain, or ischemic colitis (3%); Three died from aneurysm (1%), and 11 died of other vascular causes (4%) (dementia, generalized arteriosclerosis), compared with 93 women (36%) from the corresponding causes: 35 (13%), 27 (10%), 14 (5%), 4 (2%), and 13 (5%), respectively. Nonvascular causes of death occurred in 55 men (19%) versus 37 women (14%) as follows: uraemia in 4 and 3 cases (1% and 1%), pneumonia in 9 and 11 (3% and 4%), pulmonary insufficiency in 4 and 1 (1% and 0.4%)), cancer in 26 and 13 (9% and 5%), sepsis, pyelonephritis, or cholecystitis in 7 and 3 (2% and 1%), poisoning or chronic alcoholism in 4 and 1 (1% and 0.4%), and other causes or duodenal ulcer in 1 and 5 (0.3% and 2%) (Causa mortis ignota 2, pulmonary embolism + temporal arteritis 1, ileus 1, diarrhea 1), respectively.

### 3.7 Mortality with regard to diagnosis and sex in the Swedish population

**Figure 3:**
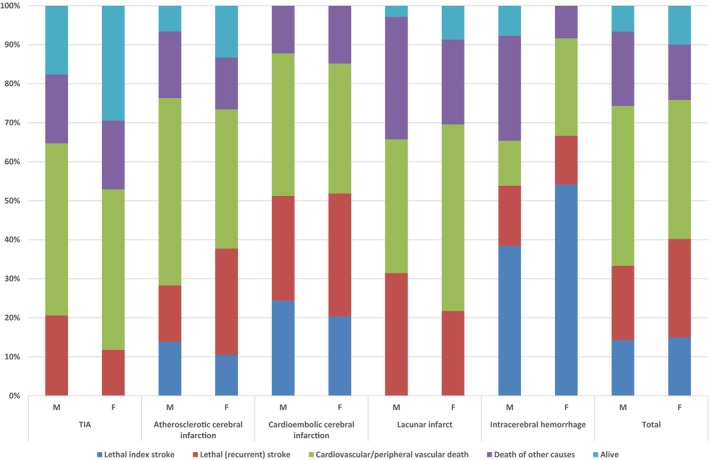
Alive and all‐cause mortality depending on diagnosis in men and women during the 25‐year follow‐up study

**Figure 4:**
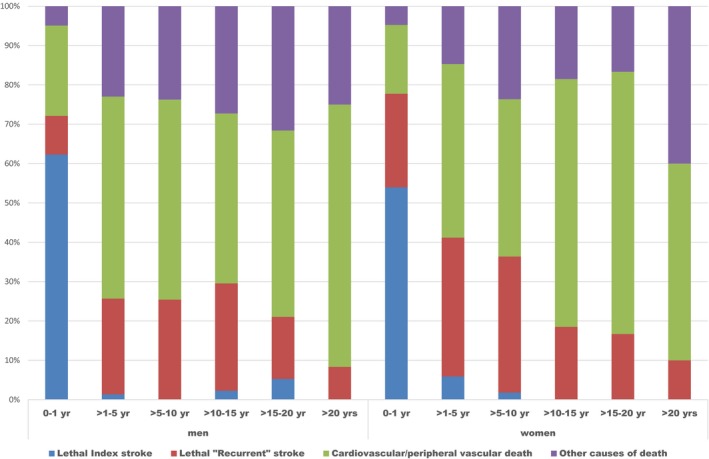
Causes of death during different intervals in men (*n *= 269) and women (*n *= 235)

During the observation period, 269 (93%) men died versus 235 (90%) women. Kaplan–Meier estimates were calculated of the probabilities of survival for each diagnosis during the observation period in men and women (Table 5). At one year, the mortality rate for men was 21.8% (ICH 34.6%, ACI 23.6%, and CE 39%) versus 23.4% in women (ICH 45.8%, ACI 20.9%, and CE 29.6%). Living patients and causes of death according to diagnosis and sex are shown in Figure 3, whereas the distribution of deaths over the long‐term observation period is shown in Figure 4.

**Table 5:** Kaplan–Meier estimates of probabilities of survival after TIA/index stroke, 1986–2011

Time after admission	Survival in each subgroup and gender. 95% CI											
	TIA		ACI		CE		LI		ICH		Total	
	M	F	M	F	M	F	M	F	M	F	M	F
I month	100	100	89 (83.8–93.5)	91 (86.2–95.6)	73 (59.7–86.7)	85 (75.8–94.6)	100	100	65 (47.2–83.6)	62 (43.1–81.9)	87 (83.1–90.9)	88 (84.1–91.9)
3 months	100	100	84 (78.3–90)	86 (80.3–91.7)	71 (56.8–84.6)	78 (66.7–88.9)	100	96 (87.3–100)	65 (47.2–83.6)	58 (38.5–78.1)	84 (79.9–88.1)	83 (78.5–87.5)
6 months	100	100	83 (76.2–88.2)	83 (77.1–89.3)	71 (56.8–84.6)	76 (64.6–87.2)	100	91 (79.7–100)	65 (47.2–83.6)	50 (30.1–69.9)	83 (78.7–87.3)	80 (75.1–84.9)
1 year	100	100	76 (69.7–82.9)	79 (72.3–85.5)	61 (46.1–75.9)	70 (58.3–82.5)	100	83 (67.1–98.1)	61 (42.9–80.1)	46 (25.9–65.9)	78 (73.3–82.7)	76 (70.9–81.1)
2 years	94 (86–100)	82 (64.4–100)	70 (63.2–77.6)	72 (64.6–79.4)	54 (38.4–69)	59 (46.2–72.4)	91 (82.2–100)	78 (61.5–95.1)	61 (42.9–80.1)	46 (25.9–65.9)	73 (68–78)	68 (62.5–73.7)
5 years	71 (55.4–85.4)	71 (48.9–92.3)	52 (44–60)	52 (44.2–60.6)	34 (14.5–48.5)	35 (22.5–47.9)	66 (50–81.4)	74 (55.9–91.9)	38 (19.9–57.1)	29 (11–47.4)	52 (46.3–57.7)	50 (43.9–56.1)
10 years	53 (36–69.7)	59 (35.5–82.1)	31 (23.9–37.9)	30 (22.7–37.5)	17 (5.5–28.7)	11.1 (2.7–19.5)	37 (21–53.1)	52 (31.9–72.5)	35 (16.4–52.8)	21 (4.7–37.1)	33 (27.5–38.5)	29 (23.5–34.5)
15 years	38 (22–54.4)	47 (23.4–70.8)	17 (11.1–23.1)	20 (13.6–27)	2 (0–6.7)	4 (0–8.7)	9 (0–17.8)	34.8 (15.4–54.2)	23 (6.9–39.3)	4 (0–12.2)	17 (12.7–21.3)	18 (13.3–22.7)
20 years	23 (9.2–37.8)	41 (17.9–64.5)	12 (7.5–17.5)	17 (10.7–22.9)	0	0	3 (0–8.4)	22 (4.9–38.5)	11 (0–23.8)	0	11 (7.5–14.5)	14 (9.9–18.1)
Total	18 (4.9–30.3)	29 (7.7–51.1)	7 (2.6–10.6)	13 (7.8–18.8)	0	0	3 (0–8.4)	9 (0–20.2)	8 (0–17.9)	0	7 (4.1–9.9)	10 (6.3–13.7)

Outcome over the long term were significantly different among included subgroups (log rank test): men TIA vs. ACI, *p *= .006; TIA vs. CE, *p *= .000; TIA vs. LI, *p *= .02; TIA vs. ICH, *p *= .032; CE vs. ACI, *p *= .002; LI vs. CE, *p *= .001, women TIA vs. ACI, *p *= .04; TIA vs. CE, *p *= .000; TIA vs. ICH, *p *= .000; CE vs. ACI, *p *= .000; ICH vs. ACI, *p *= .002; CE vs. LI, *p *= .000; ICH vs. LI, *p *= .001.

Of the included patients with DM at admission, the mean age was 71.4 years, and 2% of the men survived; in women, the mean age was 73.2 years, and 0% survived. In men with increased fasting blood glucose (DM not known), the mean age was 72, compared to; 5%, in women with a mean age of 75.4; 7%. Of men with no diabetes the mean age was 68.4, compared to; 8%, in women with a mean age of 70.1; 15%. The differences between the groups existed mainly due to death from index stroke: known diabetes mellitus occurred in 15% of men and 19% of women; increased fasting blood glucose in 32% of men and 24% of women; and 8% and 9% of men and women, respectively, had no diabetes. Stroke during the observation period was more common than MI among the survivors: men, 63% versus 16% (*p *= .003); and women, 42% versus 19%, (*p *= .071). At the last follow‐up, the risk of death due to cardiosclerosis, heart failure, or arrhythmias was higher in men than in women (*p *= .014). Fatal cancer occurred in 26 men (known cancer before admission in 6 cases) (9%) versus 13 women (known cancer before admission in 3 cases) (5%) (*p *= .069**).**

**Figure 5:**
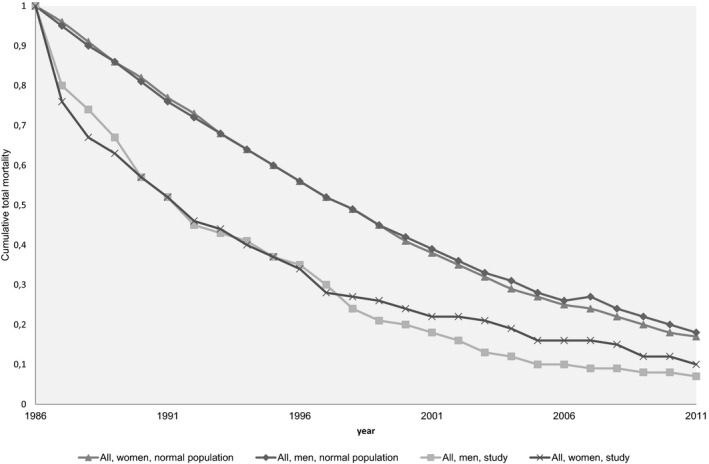
Kaplan–Meier estimates for the risk of death in all men and women over a period of 25 years in comparison with the normal population (adjusted for 2007 due to longer survival of the Swedish population)

The differences in survival among all of the included patients compared with the normal population was: in men after one year, 15%; after 10 and 15 years, 21%; after 20 years, 16%; after 25 years, 11%, versus in women 20%, 22%, 16%, 9%, and 7%, respectively (Figure 5). At 25 years follow‐up the differences of survival between diagnosis and the normal population for men were: TIA, 12% (0.3 vs. 0.18); ACI, 9% (0.16 vs. 0.07); CE, 6% (0.06 vs. 0); lacunar infarct (LI), 9% (0.12 vs. 0.03) and ICH, 20% (0.28 vs. 0.08). Women patients admitted due to TIA had even better prognosis compared with the normal population 2% (0.27 vs. 0.29), but not the others: ACI, 11% (0.24 vs. 0.13); CE, 5% (0.05 vs. 0); LI 9% (0.18 vs. 0.09) and ICH, 10% (0.1 vs. 0).

### 3.8 Outcomes during secondary prophylactic treatment

Total observation time for 288 men was 2254 years (mean 7.8 years) and for 261 women 1984 years (mean 7.6 years). In men, the distribution of AC (warfarin treatment) was 18%, compared to APT in 54%, no treatment in 27%, and unknown in 2%; in women, the percentage on AC was 15%, compared to APT in 60%, no treatment in 23%, and unknown in 2%. Table 6 provides data about the number of patients and the cumulative annual risks of stroke and MI during different observation intervals depending on diagnosis, sex, and type of treatment.

**Table 6:** The cumulative risk of all strokes/myocardial infarctions per 100 patient‐years in men and women in the different subgroups reported as total and with regard to treatment administered over different observation intervals. Observation time of APT in combination AC + APT has been included in the total observation time of APT

	Number of patients within intervals	Cumulative risk per 100 patient years	Men										Women									
			30 days	90 days	180 days	1 year	5 years	10 years	15 years	20 years	>20 years Total	Total number of patients	30 days	90 days	180 days	1 year	5 years	10 years	15 years	20 years	>20 years Total	Total number of patients
TIA	Total, *n*		34	34	34	34	34	25	18	13	8	34	17	17	17	17	17	12	10	8	7	17
	Stroke		35.8	11.9	17.9	11.8	10.6	8.2	8.4	8.1	8		71.4	47.7	23.9	11.8	2.8	3.1	4	4.8	5	
	MI		0	0	0	0	4.6	4.7	4.2	5.7	5.5		0	0	23.9	23.5	8.4	6.2	5.8	4.8	4.2	
	AC, *n*		28	26	19	13	11	7	7	4	4	31	7	7	2	1	2	2	1	2	2	10
	Stroke		45.9	16.4	18.9	12.4	15.4	15.8	11.2	8.8	7.2		0	0	0	0	0	13.4	18.2	11.3	7.4	
	MI		0	0	0	0	3.1	9	6.4	7.5	6.1		0	0	0	0	15.4	13.4	18.2	11.3	7.4	
	APT, *n*		8	12	12	20	25	18	12	8	4	29	9	12	12	13	14	11	8	6	4	17
	Stroke		0	0	23.7	16.1	12.5	8.8	9.5	9.7	9.8		0	46.7	19.6	8.6	3.5	1.9	2.8	2.9	3.7	
	MI		0	0	0	0	6.8	5.1	4.5	6.6	6.6		0	0	39.1	34.2	8.8	6.5	5.6	4.7	4.3	
	Untreated, *n*		2	2	7	8	11	6	6	1	2	16	2	3	3	3	3	2	1	2	3	8
	Stroke		0	0	0	0	9.5	5.3	6.8	6.4	6.4		1052	178.6	76.9	35.5	11.9	7.2	5.3	13.4	11.6	
	MI		0	0	0	0	3.2	1.8	2.7	2.5	2.6		0	0	0	0	0	0	0	0	0	
	Unknown, *n*		0	0	0	0	0	0	0	0	0	0	0	0	0	0	0	0	0	0	0	0
	Stroke		0	0	0	0	0	0	0	0	0		0	0	0	0	0	0	0	0	0	
	MI		0	0	0	0	0	0	0	0	0		0	0	0	0	0	0	0	0	0	
ACI	Total, *n*		152	136	129	126	116	79	47	26	19	152	143	130	123	119	113	75	43	29	24	143
	Stroke		70.2	33.4	21.8	15.2	10.6	8.9	8.5	8	7.7		63.7	41	29.4	25.8	16.1	13.8	12.3	11.3	10.5	
	MI		8.8	6.1	10.9	8.8	7.8	7.2	6.4	6.4	6.4		18.2	15.8	14.7	10.8	5.5	5.2	5.8	5.3	5.3	
	AC, *n*		67	56	38	28	19	15	7	7	5	74	56	47	27	25	21	6	6	5	4	66
	Stroke		43.4	15.8	9.5	9.6	5.4	5	4.6	4.6	4.1		53.1	38.9	30.5	23.2	24.2	19.2	18	15.1	12.9	
	MI		0	0	9.5	9.6	6.8	8.3	7.2	7.4	7.1		0	0	6.1	3.9	3.5	5.1	9.6	8	7.6	
	APT, *n*		58	63	67	76	79	51	35	18	13	110	57	65	65	72	77	51	29	22	19	105
	Stroke		75	39.8	28.6	14.7	13.6	11.6	9.7	9.0	8.7		66.2	43.7	35.3	31.5	15.4	13.2	11.6	10.9	10.2	
	MI		25	8	10.7	8.2	9.1	7.5	6.7	6.7	6.8		0	7.3	10.6	11.6	3.8	3.4	4	3.8	3.8	
	Untreated, *n*		47	39	32	39	57	32	10	9	5	100	44	35	31	31	43	22	9	9	4	81
	Stroke		108.3	52.2	27.2	22.5	9.0	8.0	9.4	9.2	8.9		74.3	41.6	20.6	28	19.5	16.4	14.4	13.3	12.5	
	MI		0	13	13.6	9.6	6.4	6.8	6.4	6	5.8		74.4	55.4	27.5	17.5	11.2	10.9	10.3	9.6	9	
	Unknown, *n*		0	1	2	3	6	2	0	0	0	8	0	4	7	8	12	3	0	1	0	13
	Stroke		0	0	0	0	0	0	0	0	0		0	0	0	0	2.9	2.3	2.3	2.2	2.2	
	MI		0	0	0	0	5	3.2	3	3	3		0	0	46.7	32.3	8.8	7	7	6.5	6.5	
CE	Total, *n*		41	30	29	29	25	15	7	2	0	41	54	47	42	41	38	19	5	2	0	54
	Stroke		0	12.9	27.2	25.2	18.8	18.8	18.3	17.9	17.9		48.3	52.6	37.1	21.7	19.8	17.4	15.7	15.7	15.7	
	MI		72.7	25.8	13.6	10.8	4.7	3.2	3	2.9	2.9		0	0	4.6	2.4	3.4	3.6	3.2	3.1	3.1	
	AC, *n*		10	11	12	11	9	6	3	0	0	14	21	18	18	16	14	10	3	2	0	25
	Stroke		0	39.5	19.2	41.4	30.6	29.5	29.3	29.3	29.3		65.8	25	11.8	6.1	16.7	15.5	13.5	12.7	12.7	
	MI		0	0	0	0	0	0	0	0	0		0	0	0	0	0	2.4	2.1	2	2	
	APT, *n*		15	13	10	13	11	6	4	2	0	19	21	22	17	19	23	8	2	0	0	33
	Stroke		0	0	56.8	29.1	14.5	15.1	14.2	13.7	13.3		0	22.1	36.1	18.8	22.9	18	16.5	16.5	16.5	
	MI		99	35.1	18.9	9.8	7.3	4.5	3.9	3.8	3.8		0	0	12.1	6.3	6.5	4.8	4.4	4.4	4.4	
	Untreated, *n*		18	9	8	7	8	5	0	1	0	22	18	14	9	11	11	3	0	1	0	27
	Stroke		0	0	0	0	11.2	14.4	14.4	14.0	14.0		90.9	138.9	78.4	50	20.9	21.7	21.7	24.6	24.6	
	MI		108.7	46.5	25.3	28	7.5	5.8	5.8	5.6	5.6		0	0	0	0	3.5	3.1	3.1	3.1	3.1	
	Unknown, *n*		0	0	1	1	0	1	0	0	0	1	0	0	0	0	0	0	0	0	0	0
	Stroke		0	0	0	0	0	0	0	0	0		0	0	0	0	0	0	0	0	0	
	MI		0	0	0	0	0	0	0	0	0		0	0	0	0	0	0	0	0	0	
LI	Total, *n*		35	35	35	35	35	23	13	3	1	35	23	23	22	21	19	16	12	8	5	23
	Stroke		0	11.6	5.8	2.9	9.4	10.6	11	10.7	10.6		52.9	53.2	27.3	19.1	6.5	4.3	4.4	4.5	4.2	
	MI		0	0	5.8	2.9	2.7	3.8	5.9	5.7	5.6		0	0	18.2	14.4	3.2	2.5	3.4	2.9	2.7	
	AC, *n*		18	18	9	5	4	4	4	0	1	19	8	8	4	1	3	3	4	2	1	10
	Stroke		0	0	0	0	15.4	14.4	13.8	13.8	13.5		156.3	119	85.8	70.4	17.3	9.2	6.3	8	7.5	
	MI		0	0	0	0	5.1	2.9	4.6	4.6	4.5		0	0	0	0	0	0	6.3	5.3	5	
	APT, *n*		16	19	23	26	28	17	10	3	1	31	13	13	14	15	14	10	8	6	3	19
	Stroke		0	26.2	11.2	4.6	8.3	11.2	11.6	11.2	11.1		0	0	0	7.8	3.9	2.3	3.6	3.8	3.4	
	MI		0	0	11.2	4.6	4.2	4.6	7.0	7.8	7.8		0	0	16.8	15.7	3.9	3.5	2.7	2.3	2.1	
	Untreated, *n*		3	6	7	5	12	7	3	1	1	17	3	7	6	5	9	7	4	3	1	12
	Stroke		0	0	0	0	8.8	6.1	7.1	7	6.8		0	76.3	36.5	18.9	6.7	5.7	4.6	4.2	4.1	
	MI		0	0	0	0	2.9	2	1.8	1.8	1.7		0	0	36.5	18.9	3.4	1.9	3.1	2.8	2.7	
	Unknown, *n*		0	0	0	0	0	0	0	0	0	0	0	0	0	0	0	0	0	0	0	0
	Stroke		0	0	0	0	0	0	0	0	0		0	0	0	0	0	0	0	0	0	
	MI		0	0	0	0	0	0	0	0	0		0	0	0	0	0	0	0	0	0	
ICH	Total, *n*		26	17	17	17	17	11	9	6	3	26	24	13	13	11	11	7	5	1	0	24
	Stroke		0	0	0	5.8	4.1	5.0	5.8	5.6	6.8		0	0	0	0	1.9	6	6.5	6.3	6.3	
	MI		0	0	11.8	5.8	4.1	2.5	3.2	2.8	2.6		0	0	0	0	0	1.2	1.1	2.1	2.1	
	AC,*n*		0	0	0	0	0	0	1	0	0	1	0	0	0	0	0	0	0	0	0	0
	Stroke		0	0	0	0	0	0	0	0	0		0	0	0	0	0	0	0	0	0	
	MI		0	0	0	0	0	0	0	0	0		0	0	0	0	0	0	0	0	0	
	APT, *n*		0	0	1	1	1	5	6	2	2	7	0	0	0	0	0	0	1	1	0	1
	Stroke		0	0	0	169.5	43.6	16.7	15	11.9	14.1		0	0	0	0	0	0	0	0	0	
	MI		0	0	0	0	21.8	16.7	9	7.2	6		0	0	0	0	0	0	0	0	0	
	Untreated, *n*		26	17	17	16	16	10	5	4	2	26	24	13	13	11	11	7	5	0	0	24
	Stroke		0	0	0	0	1.4	3	3.3	3.7	4.3		0	0	0	0	1.9	6	6.6	6.6	6.6	
	MI		0	0	11.9	6	2.9	2	1.7	1.5	1.4		0	0	0	0	0	1.2	1.1	1.1	1.1	
	Unknown, *n*		0	0	0	0	0	0	0	0	0	0	0	0	0	0	0	0	0	1	0	1
	Stroke		0	0	0	0	0	0	0	0	0		0	0	0	0	0	0	0	0	0	
	MI		0	0	0	0	0	0	0	0	0		0	0	0	0	0	0	0	87.7	87.7	

**Figure 6:**
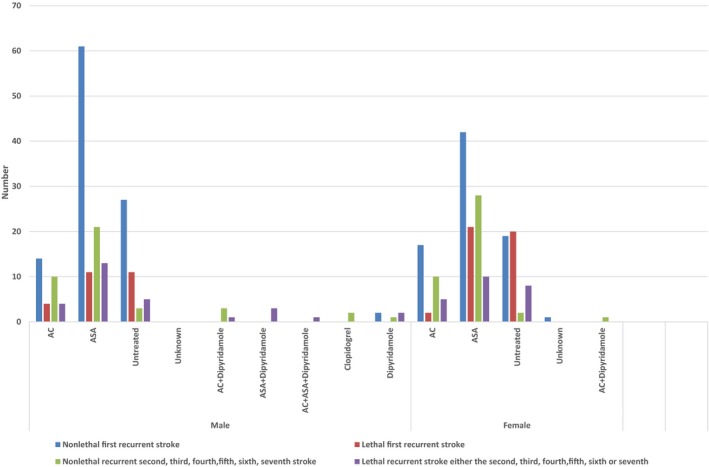
The types of treatment at the first event of stroke, as well as in later events of strokes, fatal or not

**Figure 7:**
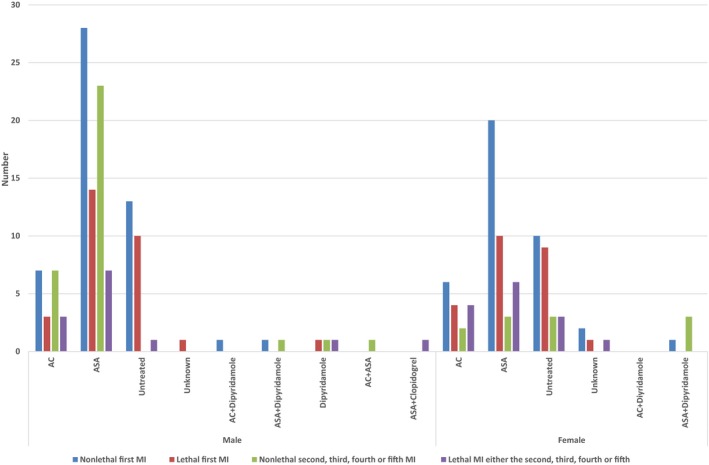
The types of treatment at the first event of myocardial infarction, as well as in later events of myocardial infarctions, fatal or not

The types of treatment at the first event of stroke/MI, as well as in later events of stroke/MI, fatal or not, are reported in Figures 6, 7.

**Figure 8:**
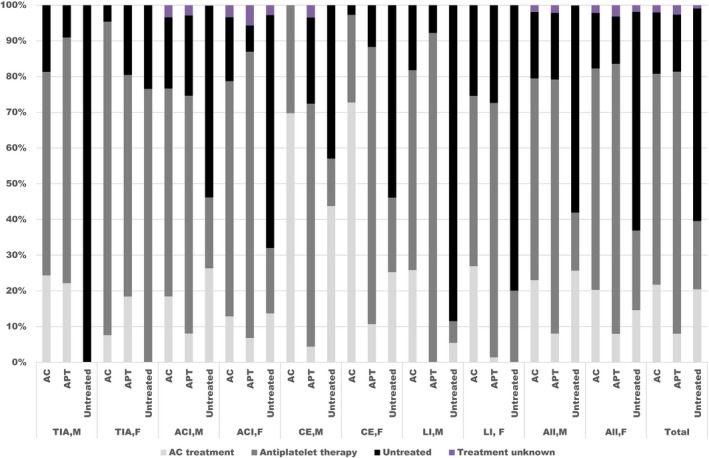
First‐line treatment and its distribution to other treatments in patients with TIA / ischemic stroke during the observation period

First‐line secondary prophylactic treatment (AC, APT, untreated) versus other treatments in patients with TIA/ischemic stroke are reported in Figure 8. A combination of AC and APT was administered to only six men and two women.

The occurrences of stroke (nonfatal, fatal) and MI (nonfatal, fatal) and different causes of death at the first‐line treatment in patients with TIA/ischemic stroke (%/yr.), and the total risk of the allocated treatments are reported in Table 7. More than 2 strokes had occurred in 22 patients admitted due to TIA/ischemic stroke. First‐line treatment was AC in 8 men compared with 7 in women, ASA in 4 men versus in one woman, and untreated in 2 men. More than 2 MIs had occurred in 10 men and 4 women, of whom first‐line treatment was AC in 7 men compared with 2 in women, ASA in 3 men and in one woman, and untreated in one woman, respectively.

**Table 7:** Events of strokes, myocardial infarctions and deaths during the observation time according to first‐line treatment and the total risk per 100 patient‐years in patients with TIA and different subgroups of ischemic stroke. Numbers within parentheses denote numbers of patients

	Men																		
	First‐line (AC)					First‐line (APT)					First‐line (Neither AC nor APT)					Total			
Number	118^1^					85^1^					60					138	191	155	9
Age (mean) years	65.1					73.3					75.9								

	Total	AC	APT^3^	Untr	Unk	Total	AC	APT^4^	Untr	Unk	Total	AC	APT	Untr	Unk	AC	APT^5^	Untr	Unk
Observation time, years	1269	292	718	236	24	583	47	427	109	12	209	54	34	121	0.3	393	1180	466	37
Stroke	7.7 (98)	9.9 (29)^a^	7.5 (54)^a^	7.2 (17)	0	10.8 (63)	10.6 (5)^b^	12.4 (53)^b^	7.3 (8)	0	12 (25)	5.6 (3)	20.6 (7)	12.4 (15)	0	9.4 (37)^c^	9.7 (114)^c^	8.6 (40)	0
Fatal	2.1 (26)	3.1 (9)^a^	2.2 (16)^a^	1.3 (3)	0	2.4 (14)	2.1 (1)	2.3 (10)	2.8 (3)	0	5.3 (11)	0	5.9 (2)	7.4 (9)	0	2.5 (10)^a^	2.4 (28)^a^	3.2 (15)	0
Nonfatal	5.7 (72)	6.9 (20)	5.3 (38)	5.9 (14)	0	8.4 (49)	8.5 (4)^b^	10.1 (43)^b^	4.6 (5)	0	6.7 (14)	5.6 (3)	14.7 (5)	5 (6)	0	6.9 (27)^b^	7.3 (86)^b^	5.4 (25)	0
Myocardial infarction	5.3 (67)	5.1 (15)^d^	6.1 (44)^d^	3.8 (9)	0	7.5 (44)	12.8 (6)^b^	8 (34)^b^	5.5 (6)	8.3 (1)	4.3 (9)	1.9 (1)	2.9 (1)	5.8 (7)	0	5.6 (22)^e^	6.7 (79)^e^	4.7 (22)	2.7 (1)
Fatal	1.5 (19)	0.7 (2)	2 (14)	1.3 (3)	0	3.1 (18)	4.3 (2)f	2.8 (12)f	3.7 (4)	8.3 (1)	2.4 (5)	1.9 (1)	0	3.3 (4)	0	1.3 (5) f	2.2 (26)f	2.4 (11)	2.7 (1)
Nonfatal	3.8 (48)	4.5 (13)^d^	4.2 (30)d	2.5 (6)	0	4.5 (26)	8.5 (4^)^ g	5.2 (22)^g^	1.8 (2)	0	1.9 (4)	0	2.9 (1)	2.5 (3)	0	4.3 (17)^h^	4.5 (53)^h^	2.4 (11)	0
Death	8.1 (103)^1^	7.9 (23)a	7.9 (57)a	9.8 (23)^1^	8.3 (2)	14.4 (84)^1^	10.6 (5)	12.9 (55)	20.2 (22)^1^	16.7 (2)	28.2 (59)	7.4 (4)	23.5 (8)	38.8 (47)	0	8.1 (32)^a^	10.3 (121)^a^	19.5 (91)^1^	10.8 (4)
Death index stroke	0.4 (5)^1^	0	0.3 (2)	1.3 (3)^1^	0	0.9 (5)^1^	0	0.7 (3)	1.8 (2)^1^	0	10.5 (22)	0	0	18.2 (22)^1^	0	0	0.4 (5)	5.6 (26)^1^	0
Fatal recurrent stroke	2.1 (26)	3.1 (9)^a^	2.2 (16)^a^	1.3 (3)	0	2.4 (14)	2.1 (1)	23 (10)	2.8 (3)	0	5.3 (11)	0	5.9 (2)	7.4 (9)	0	2.5 (10)^a^	2.4 (28)^a^	3.2 (15)	0
Cardiovascular death	4.3 (55)	4.5 (13)	4.2 (30)	4.2 (10)	8.3 (2)	7.9 (46)	6.4 (3)	7.3 (31)	9.2 (10)	16.7 (2)	7.2 (15)	3.7 (2)	11.8 (4)	7.4 (9) 1	0	4.6 (18)f	5.6 (66)f	6.2 (29) 1	10.8 (4)
Other causes of death	1.3 (17)	0.3 (1)	1.3 (9)	3 (7)	0	3.3 (19)	2.1 (1)	2.6 (11)	6.4 (7)	0	5.7 (12)	3.7 (2)	5.9 (2)	6.6 (8)	0	1 (4)	1.9 (22)	4.7 (22)	0

	Women																		
	First‐line (AC)					First‐line (APT)					First‐line (Neither AC nor APT)					Total			
Number	87					94					56					111	173	128	13
Age, mean	65.5					76.5					75.1								

	Total	AC	APT	Untr	Unk	Total	AC	APT	Untr	Unk	Total	AC	APT	Untr	Unk	AC	APT	Untr	Unk
Observation time, years	1121	227	699^6^	174	24	581	46	439	77	18	185	27	41	114	3	301	1181^6^	365	46
Stroke	8.4 (94)	11 (25)^g^	7.9 (55)^g^	9.2 (16)	0	11.4 (66)	21.7 (10)	10.3 (45)	13 (10)	5.6 (1)	11.4 (21)	0	9.8 (4)	14.9 (17)	0	11.6 (35)^g^	8.8 (104)^g^	11.8 (43)	2.2 (1)
Fatal	1.7 (19)	1.8 (4)	0.9 (6)	5.2 (9)	0	5.3 (31)	6.5 (3)	5 (22)	7.8 (6)	0	7 (13)	0	7.3 (3)	8.8 (10)	0	2.3 (7)	2.6 (31)	6.8 (25)	0
Nonfatal	6.7 (75)	9.3 (21)^g^	7 (49)^g^	4 (7)	0	6 (35)	15.2 (7)	5.2 (23)	5.2 (4)	5.6 (1)	4.3 (8)	0	2.4 (1)	6.1 (7)	0	9.3 (28)^g^	6.2 (73)^g^	4.9 (18)	2.2 (1)
Myocardial infarction	3.8 (42)	5.7 (13)^f^	3 (21)^f^	4 (7)	8.3 (2)	5.3 (31)	4.3 (2)	4.3 (19)	11.7 (9)	5.6 (1)	7 (13)	3.7 (1)	9.8 (4)	7 (8)	0	5.3 (16) f	3.7 (44) f	6.6 (24)	6.5 (3)
Fatal	1.2 (14)	2.2 (5)	0.9 (6)	1.2 (2)	4.2 (1)	2.6 (15)	4.3 (2)	1.8 (8)	6.5 (5)	0	4.3 (8)	3.7 (1)	4.9 (2)	4.4 (5)	0	2.7 (8)	1.4 (16)	3.3 (12)	2.2 (1)
Nonfatal	2.5 (28)	3.5 (8)^f^	2.1 (15)^f^	2.9 (5)	4.2 (1)	2.8 (16)	0	2.5 (11)	5.2 (4)	5.6 (1)	2.7 (5)	0	4.9 (2)	2.6 (3)	0	2.7 (8) f	2.4 (28) f	3.3 (12)	4.3 (2)
Death	6 (67)	6.6 (15)	4.3 (30)	12.1 (21)	4.2 (1)	15.5 (90)	17.4 (8)	13.7 (60)	26 (20)	11.1 (2)	29.2 (54)	7.4 (2)	22 (9)	37.7 (43)	0	8.3 (25)	8.4 (99)	23 (84)	6.5 (3)
Death index stroke	0.3 (3)	0	0	1.7 (3)	0	1 (6)	0	0.7 (3)	2.6 (2)	5.6 (1)	9.2 (17)	0	0	14.9 (17)	0	0	0.3 (3)	6 (22)	2.2 (1)
Fatal recurrent stroke	1.7 (19)^1^	1.8 (4)	0.9 (6)	5.2 (9)^1^	0	5.3 (31)^1^	6.5 (3)	5.0 (22)	7.8 (6)^1^	0	7 (13)^1^	0	7.3 (3)	8.8 (10)^1^	0	2.3 (7)	2.6 (31)	6.9 (25) 2	0
Cardiovascular death	2.8 (31)^1^	3.5 (8)	2.4 (17)	2.9 (5)^1^	4.2 (1)	6.9 (40)^1^	8.7 (4)	5.9 (26)	11.7 (9)^1^	5.6 (1)	10.3 (19)^1^	7.4 (2)	12.2 (5)	10.5 (12)^1^	0	4.7 (14)	4.1 (48)	7.1 (26) 2	4.3 (2)
Other causes of death	1.3 (15)	1.3 (3)	1 (7)	2.9 (5)	0	2.4 (14)	2.2 (1)	2.1 (9)	5.2 (4)	0	3.2 (6)	0	2.4 (1)	4.4 (5)	0	1.3 (4)	1.4 (17)	3.8 (14)	0

^1^One patient in each group. ^2^Three patients in each group.

Treatment with DP/ASA together with AC included in the observation time: ^3^0.8 years ; ^4^12 years; ^5^13 years, ^6^3.8 years.

^a^ac + dipyridamole = 1;ac + asa + dipyridamole = 1.

^b^ac + dipyridamole = 3.

^c^ac + dipyridamole = 1;ac + asa + dipyridamole = 1; ac + dipyridamole = 3.

^d^ac + asa  = 1.

^e^ac + asa = 1; ac + dipyridamole  = 3.

^f^ac + dipyridamole = 1.

^g^ac + dipyridamole  = 2.

^h^ac + dipyridamole  = 2; ac + asa  = 1.

According to the intention to treat analysis, including all of the patients with TIA/ischemic stroke, patients allocated AC had lower annual risk regarding events of strokes, MIs, and death than with APT in men and women and APT versus no treatment, except for MI in men.

Including all 549 patients, AC as the given treatment was more favorable for men regarding the annual risk of stroke, compared with APT (9.4% vs. 9.8%), as well as the risks of MI, (5.6% vs. 6.7%), and death (8.1% vs. 10.3%), compared to women for stroke (11.6% vs. 8.8%) and MI (5.3% vs. 3.7%) but not for death (8.3% vs. 8.4%).

### 3.9 Secondary prophylactic treatment and side effects, occurrences of venous thrombosis, pulmonary embolism and embolization not in the brain

Fatal bleeding occurred in 6 men, of whom 3 had bleeding on AC treatment alone (duodenal ulcer 1, ICH 1, SAH 1); in combination with APT there was one case of ICH, in 2 cases of APT ICH, as well as four cases in women (ICH on AC 2, ICH on APT 1, SAH untreated 1) (Table 8). The risk of fatal bleeding was annually 0.86% on AC compared to 0.17% on APT. Venous thrombosis was reported at a rate of 0.4% per year in men on APT versus 0.5% in women. Pulmonary embolism occurred in 8 men who were untreated (1.4% per year) versus in 12 women (2.6% per year). There were statistically significant differences between AC treatment versus no treatment regarding the risk of pulmonary embolism (*p *= .000), and between APT and no treatment (*p *= .005), but not between AC‐treated versus APT‐treated patients. In total, 86 events of venous thrombosis, pulmonary embolism, embolization (not in the brain) and/or side effects were reported in 63 men, compared to 69 reports in 55 women. The annual risks of complications reported above and side effects during AC treatment were 5.9% in men and 4.4% in women, while the corresponding values on APT in men and women were 3.7% and 3%, and in untreated men and women, 3% and 4.4%, respectively.

**Table 8:** Side effects, embolization (not brain) with and without secondary prophylactic treatment

Sex	Men			Women		
Treatment	AC	APT	Untreated	AC	APT	Untreated
Number	*N *= 139	*N *= 198	*N *= 181	*N *= 111	*N *= 174	*N *= 152
Side effects, complications	Number Annually (%/year)			Number Annually (%/year)		
Severe intracranial bleeding	8 (1^a^)^1^2	6 (1^a^)^2^0.5	2^3^0.4	5^4^1.7	1^5^0.08	2^6^0.4
Dyspepsia, gastrointestinal bleeding, anemia	8^7^2	21^8^1.7	2^9^0.4	3^10^1	16^11^1.4	2^12^0.4
Hematuria	30.8	20.2	20.4	10.3		10.2
Hematoma	51.3			31		
Epitaxis, other bleedings		40.3			30.3	
Headache, vomiting (dipyridamole)		20.2			30.3	
Allergy to ASA		40.3			20.2	
Embolization (not in the brain)		10.08				20.4

^1^vs. ^2^, ns, ^1^ vs. ^3^, *p *= .023, ^2^ vs. ^3^, ns, ^4^ vs. ^5^ ns, ^7^ vs. ^8^ ns, ^7^ vs. ^9^, *p *= .018, ^8^ vs. ^9^ , *p *= .000, ^10^ vs. ^11^, *p *= .031, ^11^ vs. ^12^ , *p *= .002.

a Within parenthesis treatment with both AC and APT.

### 3.10 Changes in AC/APT treatment and causes to no treatment

A total of 437 changes of treatment were reported in men vs. 323 in women. The mean and median number of different treatments were 2.2 and; 2 in men vs. 1.9 and; 2 in women (range 0–11 in both sexes). Causes of changes in treatment were the following: by usage (M 155, W 122), progressive stroke (M 7, W 7), stroke/TIA (M 73, W 57), MI (M 20, W 10), Swedish aspirin low dose trial (SALT‐trial) (M 7, W 6), thrombosis, pulmonary embolism, embolism not in the brain (M 11, W 16), side effects (M 45, W 24), AF (M 14, W 2), and lack of compliance and other causes (M 105, W 79).

The causes for no treatment in 155 men and 128 women with TIA/ischemic stroke were: progressive stroke (M 1, W 1), major stroke (M 39, W 35), the physicians have not prescribed further treatment (M 77, W 70), side effects of ASA (M 17, W 7), side effects of AC (M 4, W 4), compliance (M 1, W 1), SALT‐trial (M 6, W 4), recurrent stroke (M 2, W 1), and other causes (M 8, W 5). Among the 60 untreated men and 56 untreated women as first‐line treatment 27% of men and 29% among women later on received secondary prophylactic treatment compared to the others 53%, 43%, respectively.

### 3.11 Predictors of stroke, myocardial infarction and death

According to Cox regression analysis, ACI and CE versus ICH were shown to be predictors of an event of stroke (Table 9), whereas predictors for MI were: age, angina pectoris, previous MI, heart failure and diagnosis of increased risk, especially in patients with TIA and ACI versus ICH (Table 10).

**Table 9:** Predictors of a recurrent stroke^a^

Baseline characteristics	OR	*p*‐value
History of diabetes/fasting blood glucose ≥6.1 mmol/L	1.28 (0.98–1.69)	.073
Diagnosis^b^		.012
ACI	2.36 (1.29–4.3)	.005
CE	2.58 (1.34–4.96)	.005
Previous stroke	1.33 (0.99–1.78)	.058
Angina pectoris	0.67 (0.45–1.01)	.055
Hypertension/treatment with antihypertensive drugs	1.33 (0.99–1.78)	.057

OR = odds ratios; 95% confidence intervals in parenthesis.

Variables considered: age, sex, diagnosis, TIA/severity of stroke, hypertension/treatment with antihypertensive drugs, systolic/diastolic blood pressure, angina pectoris, previous myocardial infarction, heart failure, atrial fibrillation, claudication intermittens, diabetes, diabetes/fasting blood glucose ≥6.1 mmol/L, TIA previously, previous stroke and first‐line secondary prophylactic treatment (anticoagulants, antiplatelet therapy or untreated).

^a^486 cases available in analysis, 240 events, ^b^versus ICH.

**Table 10:** Predictors of MI^a^

Baseline characteristics	OR	*p*‐value
Age (per year increase in age)	1.03 (1.01–1.05)	.008
Angina pectoris	1.8 (1.15–2.81)	.010
Blood pressure, systolic (per mm Hg)	1.006 (1.0–1.012)	.039
Diagnosis^b^		.007
TIA	2.15 (0.71–6.49)	ns
ACI	2.2 (0.8–6.08)	ns
CE	0.66 (0.20–2.20)	ns
LI	1.32 (0.43–4.05)	ns
Previous myocardial infarction	1.84 (1.2–2.81)	.005
Heart failure	1.73 (1.09–2.74)	.02
TIA previously	1.47 (1–2.17)	.052

OR = odds ratios; 95% confidence interval in parenthesis.

Variables considered: age, sex, diagnosis, TIA/severity of stroke, hypertension/treatment with antihypertensive drugs, systolic/diastolic blood pressure, angina pectoris, previous myocardial infarction, heart failure, atrial fibrillation, claudication intermittens, diabetes, history of diabetes/fasting blood glucose ≥6.1 mmol/L, TIA previously, previous stroke and first line secondary prophylactic treatment (anticoagulants, antiplatelet therapy or untreated).

^a^507 cases available in analysis, 130 events, ^b^versus ICH.

According to Cox regression analysis, predictors of death among patients with TIA/ischemic stroke were age, history of diabetes/fasting blood glucose ≥6.1 mmol/L, TIA/severity of stroke versus major stroke, hypertension/treatment with antihypertensive drugs, previous MI, previous stroke, whereas first‐line secondary prophylactic treatment had beneficial effects on survival: AC OR 0.67 (0.5–0.9); ASA OR 0.67 (0.52–0.88) versus untreated. Women had a lower OR risk of death compared with men, but not statistically significant (Table 11).

**Table 11:** Predictors of death^a^ (excluding ICH)

Baseline characteristics	OR	*p*‐value
Age (per year increase in age)	1.08 (1.06–1.09)	.000
Sex^b^	0.83(0.68–1.01)	.066
History of diabetes/fasting blood glucose ≥6.1 mmol/L	1.35 (1.01–1.67)	.005
TIA/Severity^c^		.000
TIA	0.57 (0.39–0.85)	.005
Minor stroke	0.64 (0.5–0.82)	.000
Moderate stroke	0.92 (0.69–1.24)	.599
Hypertension/treatment with antihypertensive drugs	1.47 (1.17–1.85)	.001
Previous myocardial infarction	1.31 (1.04–1.65)	.023
First‐line treatment^d^		.008
AC	0.67 (0.5–0.9)	.008
Antiplatelet therapy	0.67 (0.52–0.88)	.003
Previous stroke	1.5 (1.2–1.87)	.000

OR = odds ratios; 95% confidence interval in parenthesis.

Variables considered: age, sex, diagnosis, TIA/severity of stroke, hypertension/treatment with antihypertensive drugs, systolic/diastolic blood pressure, angina pectoris, previous myocardial infarction, heart failure, atrial fibrillation, claudication intermittens, diabetes, history of diabetes/fasting blood glucose ≥6.1 mmol/L, TIA previously, previous stroke and first‐line secondary prophylactic treatment (anticoagulants, antiplatelet therapy or untreated).

^a^467 cases available in analysis, 426 events, ^b^vs. men, ^c^vs. major stroke,^d^vs. untreated.

## 4 Discussion

In this study, Kaplan–Meier analysis showed a higher risk among women than men of having a stroke during the first year −16.8% (11.8–21.8) vs. 12% (8–16) but after 15 years, it was the opposite −67.4% (55.4–75) vs. 60% (52–68) as well as after 25 years −78.9% (69.9–87.9%) vs. 70.9% (62.9–78.9%). At 5 years, the recurrence rate in men with LI was 36% (20–52%) vs. 18.4% (2.4–34.4%) in women, which can be compared with 22.4% (range 15–28%) after 4–5 years (Norrving, 2003). Log rank tests showed differences between diagnosis with an increased risk in men with CE versus TIA and ICH, as well as in women for CE versus TIA and LI and for; ACI versus LI. The risk of having a stroke in areas other than the index symptoms and signs was high in both sexes and the annual risk of stroke remained high for a very long time in both sexes. Analyses of the annual risk of stroke depending on the diagnosis and sex after 10 and after 25 years of observation, respectively, found that there was a reduction in the annual risk of stroke in men and women at index diagnoses of CE and ACI, but the annual risk of stroke was slightly increased for women with TIA and in men with ICH, while the annual risk of stroke was unchanged in men with TIA, LI, and in women with LI and ICH, respectively.

Of all strokes, 12.1% in men and 10.1% in women occurred among those patients who first had verified AF during the observation period. Few patients (1.4% men, 1.9% women) had a recurrent stroke <3 months after a MI. These figures were slightly higher than in other studies of in‐hospital stroke or during the first month (Albaker et al., 2011; Witt et al., 2006).

In this trial Cox regression analysis showed index diagnoses of CE and ACI to be statistically significant predictors of having a recurrent stroke, and other important risk factors were previous stroke, history of diabetes mellitus/fasting blood glucose ≥6.1 mmol/L or hypertension /treatment with antihypertensive drugs. Compared with the previous study (Eriksson & Olsson, 2001), where the variable diagnosis was not included in the analysis: age, severity, previous stroke and systolic blood pressure were each reported as predictors for recurrent stroke. According to intention to treat, the annual risk of stroke in patients with TIA/ischemic stroke for patients allocated to AC was 7.7% in men and 8.4% in women; with allocation to APT, the rates were 10.8% and 11.4%, and in untreated patients, they were 12% and 11.4%, respectively. Lower risks on AC versus APT were reported in men with diagnoses of TIA and ACI but not of LI or for CE patients. In women, lower risks were found on APT versus AC in patients with TIA, ACI, and LI but not in patients with CE among the TIA/ischemic stroke patients. The findings that women's responses to AC treatment were better than those of men among patients with CE were previously reported (Laupacis et al., 1994). It is not likely to assume that differences from therapeutic INR 2–3 will explain these differences (Wieloch et al., 2011). Many recurrences with unchanged treatment occurring of one and the same patient is of great importance for the interpretation of results. Another explanation could be that it was the selection of few patients with high risk among the AC‐treated men (Kim et al., 2011). The observation period of the patients with CE cerebral infarction in this trial could not be compared with NOAC trials in which the mean and median observation times were only between 1 and 2 years (Alberts et al., 2012; Diener et al., 2012; Hankey, 2014; Hori et al., 2013; Lopes et al., 2012).

The cumulative risk of a first event of MI was slightly higher in men, but the differences from women were small except for during the last 5 years of the observation period: 65.3% (43.6–87%) versus 53.3% (42.5–64.1%). Log rank tests established differences between diagnoses regarding the risk of MI, with increased risks in patients with TIA and ACI versus CE and ICH in men and in women with ACI versus CE and ICH.

The predictors of MI were age, angina pectoris, systolic blood pressure, and diagnosis versus ICH, with almost the same high risk for patients with TIA and ACI, and a lower risk for patients with LI, with the lowest risk in patients with CE stroke. Other predictors were previous MI and heart failure. According to intention to treat analysis, the annual risk of MI among men on AC was 5.3% versus 3.8% in women; on APT the rates were 7.5% and 5.3%, and without treatment they were 4.3% and 7%, respectively. The low annual risk of MI among the untreated men could partly be explained by the low frequency of MIs early during the observation period and partly by these patients later receiving AC or APT. Including all of the patients, AC was a slightly better alternative than APT in men, with annual risks of MI of 5.6% versus 6.7%, and the differences with more favorable effects for AC treatment were found in patients admitted due to TIA, CE, and LI. In women, APT was a better alternative than AC with annual risks of 3.7% versus 5.3%, and differences in favour of APT were found in patients with TIA, ACI, and LI.

Sacco and colleagues reported after ICH a case‐fatality rate of 34.6% at 7 days, which increased to 50.3% at 30 days and to 59% at 1 year, while 10‐year survival was 24.1% (Sacco, Marini, Toni, Olivieri, & Carolei, 2009). Others had reported mortality rates of 30–50% after 1 month, approximately 50% after one year and approximately 75% after 11 years (McGuire, Raikou, Whittle, & Christensen, 2007; Nilsson, Lindgren, Brandt, & Säveland, 2002). In this study, the cumulative probabilities of death in patients with ICH after 1 year, 10 years, 20 years, and 25 years are of 39%, 65%, 89%, and 92% in men versus 54%, 79%, 100% in women, respectively.

Case fatality rates due to stroke vary around the world (Feigin, Lawes, Bennett, Barker‐Collo, & Parag, 2009) and depend on the severity of stroke, the subtype of stroke and other factor (Coull et al., 2004; Eliasziw et al., 2004; Eriksson & Olsson, 2001; Evans, Harraf, Donaldson, & Kalra, 2002; Evans, Perez, Yu, & Kalra, 2000; Feigin et al., 2009; Jackson & Sudlow, 2005; de Jong, Lodder, Kessels, & van Raak, 2004; Norrving, 2003; Vaartjes, O'Flaherty, Capewell, Kappelle, & Bots, 2013; Yokota, Minematsu, Hasegawa, & Yamaguchi, 2004). Stroke mortality has decreased in many countries, but the incidence has not (Feigin et al., 2009; Vaartjes et al., 2013). Ten treatable risk factors have been associated with 90% of the risk of ischemic or hemorrhagic stroke (O'Donnell et al., 2010). The risk profile for occurrence of MI is quite different than that for stroke (Yusuf et al., 2004), but hypertension is one important risk factor for stroke, MI or vascular cause of death (Carlson & Böttiger, 1985; Conroy et al., 2003; Håheim et al., 1993; Harmsen et al., 1990; Hu et al., 2005; Psaty et al., 2001; Qizilbash, Lewington, Duffy, & Peto, 1995; Seshadri et al., 2006) and antihypertensive treatment(s) reduces these risks (Hackam & Spence, 2007; Hankey, 2014; Mancia et al., 2013; Turnbull, 2003; Turnbull et al., 2005; Wachtell, Hornestam, et al., 2005; Wachtell, Lehto, et al., 2005).

Survival after first or recurrent stroke has been reported to be <10% after 20 years, the risk factors for death were age, previous stroke, subtype of stroke, and different stroke features (Anderson et al., 2004). Lower mortality rates for patients with TIA versus stroke after one year were reported by a study in Japan after hospital discharge (7% for stroke patients and 3.5% for TIA patients) (Kimura et al., 2005). Stroke units have improved the outcomes in patients with large‐vessel infarcts but not in those with lacunar syndrome (Evans et al., 2002). In the present trial, there was also a quite different prognosis regarding survival for patients admitted due to TIA versus other ischemic subtypes of stroke after 15 years: TIA, men 38% (22–54.4%) and women 47% (23.4–70.8%); ACI, men 17% (11.1–23.1%) and women 20% (13.6–27%); CE, men 2% (0–6.7%) and women 4% (0–8.7%); and LI, men 9% (0–17.8%), and women 34.8% (15.4–54.2%). Approximately 75% of all deaths were caused by death due to index stroke, or a recurrent stroke in both sexes during the first year, thereafter percentage of a recurrent stroke as the cause of death was higher in women than in men during the first 10 years. Fatal recurrent stroke had thereafter reduced percentage impact as a cause of death, even more after 20 years, for both sexes. Another vascular cause of death was of increased percentage significance in women between 10 and 20 years compared with men, but not after 20 years.

In total, the frequency of vascular causes of death was slightly lower in men 74.3% versus women 75.9%. Women had a higher risk of death due to index stroke/recurrent stroke 40.2% versus 33.3%; ns but a lower risk of death due to cancer 5% versus 9%. In patients with increased fasting blood glucose levels/diabetes mellitus, increased mortality rates have been reported (Emerging Risk Factors Collaboration, 2011; Eriksson & Olsson, 2001) as in the present trial where the differences existed mainly for index stroke. Men had a higher risk of death due to cardiosclerosis, heart failure, or arrhythmia versus women (*p *= .014), which could have partly depended on a higher frequency of several MIs. No classification was performed at admission to assess whether the heart failure was systolic or diastolic, but the prevalence and mortality rate were reported to be almost the same (Bhatia et al., 2006; Owan et al., 2006). The clinical importance remains to be proved of classifying more MIs in women with high sensitivity cardiac troponin with a diagnostic threshold of 16 ng/L and through these means, improving outcomes due to tailored treatment (Shah et al., 2015).

The total risks of death per year, including all ICH patients, were 11.9% in men and 11.8% in women. Compared with the normal population, differences in survival increased after one year, including in all men and women. After more than 12 years in women versus 23 years in men, the differences regarding survival decreased compared with the first year and with the normal population, but there were great differences depending on TIA/subgroup of stroke. Including all patients' survival curves after 15 years showed higher mortality rate in men compared to women.

The previous study (Eriksson & Olsson, 2001) that did not include the variable secondary prophylactic treatment but the patient group with ICH in Cox regression analysis found that age, stroke severity, previous stroke, heart failure, diabetes/fasting blood glucose ≥6.1 mmol/L were each to be important predictors of death. In this study heart failure had been replaced by previous MI and hypertension as predictors but the other predictors were the same: this as the previous study supported adequate treatment for many risk factors for vascular death, both as primary and secondary prevention.

Cox regression analysis and intention to treat analysis, as analyses of events on given treatments provided support for secondary prophylactic treatment, either with AC or ASA 75 mg once daily, over the long‐term observation period unless there were contraindications.

In this trial, AC treatment seemed to have been more favorable than APT in men, whereas women had similar or better effects of APT versus AC except for regarding CE cerebral infarction. In contrast, the risk of severe bleedings on AC treatment was higher than with no treatment (*p *= .023). Gastrointestinal bleeding is a common side effect of APT (Antithrombotic Trialists' Collaboration, 2009) and in the present trial, the annual risks either dyspepsia, gastrointestinal bleeding, or anemia were considerably higher with AC or ASA versus no treatment. Treatment with AC and APT combined increased the risk of severe ICH (Toyoda et al., 2009) and the intensity of anticoagulation (Blackshear et al., 1996; Connolly et al., 2008; Hylek et al., 2003). Åsberg, Henriksson, Farahmand, and Terént (2013) reported annual hemorrhage rates of 2.5% on AC versus 2.4% on APT, with increasing risk of fatal hemorrhage for both gastrointestinal bleeding and intracranial bleeding with increasing age, whereas this trial found great differences in the annual risk of fatal bleeding between the different types of treatment.

Due to the verified beneficial effects of NOACs in patients with AF compared with warfarin (Alberts et al., 2012; Diener et al., 2012; Hankey, 2014; Hori et al., 2013; Lopes et al., 2012) and the observation in the present trial of a higher annual risk of death during treatment with APT versus AC in men compared with women, this trial supported studies evaluating NOACs, especially in male patients with arterial thromboembolism or myocardial infarction.

Randomized trials have their limitations due to the selection of patients (Maasland et al., 2009) and they usually have observation periods of <5 years. In observational studies there are also major sources of bias, especially in the selection of patients given secondary prophylactic treatment/the type of treatment or not; in contrast the observation time could be much longer and observational studies are important complements to randomized trials (MacMahon & Collins, 2001). Selection of the patients to be included in an observational study of hospitalized patients is of importance, especially regarding patients with TIA (Prabhakaran, Silver, Warrior, McClenathan, & Lee, 2008). Many patients with “TIA” at admission were therefore not included in this trial. Another limitation of the study was that many risk factors were not analyzed, especially smoking habits (Carlson & Böttiger, 1985; Conroy et al., 2003; Eriksson, 1985; Hackam & Spence, 2007; Håheim et al., 1993; Harmsen et al., 1990; O'Donnell et al., 2010; Wilhelmsen, Dellborg, Welin, & Svärdsudd, 2015; Yusuf et al., 2004).

## Conflict of Interests

None.
